# Effective Activation of Peroxymonosulfate by Oxygen Vacancy Induced Musa Basjoo Biochar to Degrade Sulfamethoxazole: Efficiency and Mechanism

**DOI:** 10.3390/toxics12040283

**Published:** 2024-04-12

**Authors:** Shuqi Li, Jian Yang, Kaiwen Zheng, Shilong He, Zhigang Liu, Shuang Song, Tao Zeng

**Affiliations:** 1 School of Environment and Spatial Informatics, China University of Mining and Technology, Xuzhou 221116, China; lishuqi@zjut.edu.cn (S.L.); hslongrcees@163.com (S.H.); 2 Ecology and Health Institute, Hangzhou Vocational & Technical College, Hangzhou 310000, China; 15215821030@163.com (J.Y.); 15117965820@163.com (K.Z.); 3 Ningbo Water & Environment Group, Ningbo 315100, China; 4 Key Laboratory of Microbial Technology for Industrial Pollution Control of Zhejiang Province, College of Environment, Zhejiang University of Technology, Hangzhou 310032, China; ss@zjut.edu.cn

**Keywords:** Musa basjoo biochar, peroxymonosulfate activation, sulfamethoxazole degradation, defect/oxygen vacancy, water treatment

## Abstract

Biochar materials have garnered attention as potential catalysts for peroxymonosulfate (PMS) activation due to their cost-effectiveness, notable specific surface area, and advantageous structural properties. In this study, a suite of plantain-derived biochar (MBB-400, MBB-600, and MBB-800), possessing a well-defined pore structure and a substantial number of uniformly distributed active sites (oxygen vacancy, OVs), was synthesized through a facile calcination process at varying temperatures (400, 600, and 800 °C). These materials were designed for the activation of PMS in the degradation of sulfamethoxazole (SMX). Experimental investigations revealed that OVs not only functioned as enriched sites for pollutants, enhancing the opportunities for free radicals (^•^OH/SO_4_^•−^) and surface-bound radicals (SBRs) to attack pollutants, but also served as channels for intramolecular charge transfer leaps. This role contributed to a reduction in interfacial charge transfer resistance, expediting electron transfer rates with PMS, thereby accelerating the decomposition of pollutants. Capitalizing on these merits, the MBB-800/PMS system displayed a 61-fold enhancement in the conversion rate for SMX degradation compared to inactivated MBB/PMS system. Furthermore, the MBB-800 exhibited less cytotoxicity towards rat pheochromocytoma (PC12) cells. Hence, the straightforward calcination synthesis of MBB-800 emerges as a promising biochar catalyst with vast potential for sustainable and efficient wastewater treatment and environmental remediation.

## 1. Introduction

Emerging pollutants (EPs), encompassing pharmaceuticals and personal care products, are widely distributed in aquatic environments, posing substantial ecological hazards on a global scale [1,2,3]. Among these contaminants, sulfamethoxazole (SMX), a member of the sulfamethoxazole class, is extensively utilized in combatting various microbial-induced diseases [4,5,6]. SMX exhibits generally limited metabolic breakdown, and its environmental residues can contribute to the emergence of resistance genes and superbugs, thereby posing a substantial threat to ecosystems and human health. Concentrations of SMX in wastewater treatment facilities have been documented to span from ng L^−1^ to μg L^−1^, with untreated hospital and pharmaceutical effluents registering concentrations reaching levels as high as mg L^−1^ [7,8,9,10]. Despite advancements in wastewater treatment, conventional processes have proven inadequate in effectively removing antibiotics [11]. Hence, there is an urgent need for the development of water treatment technologies that are both efficient and environmentally sustainable.

Presently, advanced oxidation processes (AOPs) that leverage oxidant activation, such as persulfatemonosulfate (PMS), persulfatedisulfate (PS), and O_3_, are recognized as promising methodologies in wastewater treatment, which is attributed to the generation of potent oxidizing radicals (SO_4_^•−^ at 2.5~3.1 V, ^•^OH at 1.9~2.7 V) during the reaction process [12,13,14]. These processes exhibit exceptional performance in the degradation of organic pollutants, positioning them as some of the most promising technologies in the realm of wastewater treatment [15,16,17]. Polymers derived from the synthesis of chemical precursors, encompassing transition metal semiconductors (e.g., Co, Cu, Ag, Mn), have been utilized as multiphase catalysts to activate PMS. These catalysts offer notable advantages, including high activation efficiency and ease of operation. Nevertheless, a considerable subset of these polymers encounters challenges, including high cost, reduced yield, susceptibility to secondary pollution, impractical recycling techniques, and assorted limitations. These factors collectively pose significant constraints on their widespread utilization [18,19]. In relative terms, biomass, as the most prevalent renewable organic carbon source in nature, inherently embodies a distinct porous architecture and an extensive array of functional groups. These attributes serve as inherent advantages in the development of biochar-based catalysts [20]. Among many biochar materials, lignin stands out as a molecular polymer defined by a polyphenolic structure, which is one of the most frequent compounds in plants, and possesses the characteristics of low cost and renewability [21,22,23]. Under hypoxic conditions, these chemicals can undergo pyrolysis or carbonization processes, resulting in the production of valuable biochar [24]. Simultaneously, biomass experiences unique microstructural modifications as a result of its various roles, and different biochar materials inherit the remarkable structures of their respective ancestors, resulting in a variety of properties. Furthermore, through precise high-temperature calcination, defect-rich active sites can be created without the use of additional N/O components. This technique improves pollutant adsorption while also accelerating electron migration and transformation [25,26,27,28]. Hence, it is imperative to identify a suitable biomass precursor capable of facilitating stable, efficient, and economically feasible removal of emerging pollutants (EPs) through a streamlined and rapid activation process.

Musa basjoo is a perennial herbaceous plant that exhibits a fibrous pseudostem that develops from its leaf sheaths’ increased thickness. A sizable amount of naturally occurring lignocellulose with a stiff matrix structure is present in this structure. The unstructured but well-structured arrangement guarantees a large specific surface area and a rich pore structure. Additionally, the microfibril angle optimizes the fibrous material’s tension and elastic modulus by acting as a flexible modulator. Expanding on these features, Musa basjoo presents itself as a lignocellulosic precursor with significant promise [29]. But for large molecules of difficult-to-degrade EPs (like antibiotics, dyes, and endocrine disruptors), the inactivated Musa basjoo shows remarkably weak adsorption and activation capacities. This is because of its highly symmetric structure and pristine elemental sites, which prevent the formation of an induced electron delocalization effect. Furthermore, this inhibition covers the transfer of electrons, the production of reactive species, and the adsorption of oxidizing agents. Therefore, it makes sense to assume that successfully activating plantain’s surface or interface would be essential to addressing these shortcomings and positioning it as a strong contender for use as the perfect biomass material for breaking down organic contaminants.

In this work, biochar materials made from Musa basjoo with tunable defect/oxygen vacancy (OVs) quantities were designed for the first time. The degradation of different EPs was examined when PMS was activated. It was evident from characterization and experimentation tests that the OVs generated following activation effectively regulated the electronic delocalization in the surrounding area. Intramolecular charge transfer jumps were facilitated, and carrier mobility was increased. Additionally, OVs served as high-energy adsorption sites for EPs enrichment, enabling the produced reactive oxygen species (ROS) to approach the contaminants closely and increase the likelihood of molecular assaults. With these merits, a 5-fold improvement of PMS conversion was found in MBB-800/PMS system as compared with corresponding MBB/PMS system, and the overall SMX removal increased by 61 times. Furthermore, mechanistic studies showed that this system primarily utilized the breakdown pathways of surface-bound radicals (SBRs) and ^•^OH/SO_4_^•−^ to effectively oxidize a variety of organic molecules, and the MBB-800 exhibited less cytotoxicity towards rat pheochromocytoma (PC12) cells.

## 2. Materials and Methods

### 2.1. Chemicals and Reagents

Detailed information for all chemicals and reagents is provided in Supporting Information.

### 2.2. Synthesis

#### 2.2.1. Synthesis of MBB

We used freeze-drying (−65 °C, 24 h) to turn fresh Musa basjoo tissue into aerogel in a conventional synthesis. After that, the resulting aerogel was cleaned to eliminate dissolved contaminants until the pH reached 7 using ethanol and deionized water.

#### 2.2.2. Synthesis of MBB-x

Specifically, fresh Musa basjoo tissue was freeze-dried for 24 h at −65 °C. The resulting aerogel was then pre-carbonized for 1.5 h at 300 °C with a heating rate of 5 °C min^−1^ in a nitrogen atmosphere (1.0 m^3^/h sweep gas flow rate). The black result was then evenly combined with an aqueous solution of 0.1 M NaOH (NaOH and carbon in a weight ratio of 1.5:1).

The black mixture was then dehydrated at 85 °C for 5 h to eliminate moisture and ensure thorough mixing of NaOH with carbon. Subsequently, the amalgamation was transferred into a crucible and heated at 400 °C carbonization temperature for 1.5 h in nitrogen atmosphere (1.0 m^3^/h sweep gas flow rate) of 5 °C min^−1^. Then, the resulting powder underwent washing with a 0.1 M HCl aqueous solution to eliminate any residual inorganic impurities, followed by rinsing with distilled water until the pH reached 7.0. Finally, the acquired samples were dried at 60 °C under pressure overnight and labeled as MBB-400. Changing the carbonization temperature of MBB-x to 600 °C and 800 °C, the produced samples were labeled as MBB-600 and MBB-800, respectively. 

### 2.3. Characterization

Detailed information of the characterization device is provided in the Supporting Information.

### 2.4. Experimental Procedure and Analyses

The catalytic efficacy of all samples was assessed through the degradation of EPs. In a standard procedure, a catalyst (0.1 g L^−1^) was dispersed in a SMX solution (1 mg L^−1^). The reaction commenced with the addition of PMS (0.33 mM) at an initial pH of 6.8, followed by stirring the solution at 500 r min^−1^. Periodically, 1.0 mL of the reaction solution was obtained and filtered using a 0.45 μm water phase filter to separate the supernatant from the catalysts for subsequent analysis. To identify the active species in the catalytic process, phenol, TBA, MeOH, p-BQ, and FFA were introduced into the SMX solution to capture hydroxyl radicals (^•^OH), sulfate radicals (SO_4_^•−^), superoxide radicals (O_2_^•−^), and singlet oxygen (^1^O_2_), respectively. Liquid chromatography/mass spectrometry (LC/MS) was employed to characterize the intermediates formed during the SMX degradation.

### 2.5. Cell Viability Assay 

Cell viability was assessed employing the Cell Counting Kit-8 (CCK8) assay. Cells were seeded onto 96-well plates and incubated for a duration of 24 h. Subsequently, the cells were subjected to varying concentrations of 1% MeOH or 5 µg mL^−1^, 10 µg mL^−1^, 50 µg mL^−1^, and 100 µg mL^−1^ for an additional 24 h period. Following exposure, each well received 10 μL of CCK-8 solution, and the plate was incubated at 37 °C for 30 min. The optical density at 450 nm (OD450) was then determined using a microplate reader. The outcomes were expressed as the percentage of viable cells relative to the control group cell count.

## 3. Results and Discussion

### 3.1. Morphology and Structure Characterization of MBBs

**Figure 1:**
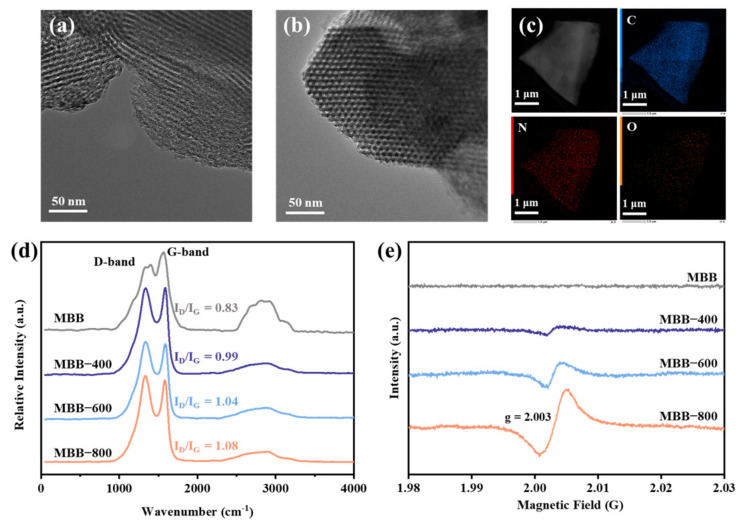
(**a**,**b**) The TEM images and (**c**) elemental mapping of MBB-800. (**d**) Raman spectra, and (**e**) EPR spectra of different samples.

Musa basjoo, abundant in lignocellulosic material, is capable of revealing varying contents of active sites after undergoing pyrolysis at different temperature profiles. TEM images unveiled that the pseudo stem of MBBs (Figure 1a,b and Figure S1) exhibited a rich and well-organized three-dimensional mesoporous tubular structure (diameter approximately at 10 nm). Notably, the tubular architecture of MBBs remained steadfast as the thermal-activated temperature increased from 400 to 800 °C, indicating that the calcination temperature exerted negligible influence on the overarching structure of MBBs [30]. Furthermore, the EDS mapping illustrated all the elements were evenly distributed in MBB-800 (Figure 1c) [31]. The specific surface area (S_BET_) of MBBs was delineated through the N_2_ adsorption–desorption isotherm (Figure S2a), which exhibited a noteworthy augmentation in the S_BET_ of thermal-activated MBB-x compared to pristine MBB, and the enhancement of S_BET_ was observed to escalate with rising temperatures. In addition, the open hysteresis loop type H2 of MBB-x demonstrated the narrow porous cylindrical structure in the range of 3–5 nm, which inhibited the desorption of N_2_ [32,33]. Furthermore, the pore size distribution curves validated the emergence of microporous structures in MBB-x due to high-temperature activation, as opposed to MBB (Figure S2b), and the specific changes in parameters were displayed in Table S1 [34]. This transformation not only enhances the adsorption of emerging pollutants (EPs) but also facilitates the exposure of additional active sites for surface catalytic reactions, thereby establishing favorable conditions for the subsequent activation of PMS [35].

To compare the fundamental physicochemical properties of various MBB samples, the crystallinity of MBBs before and after activation was initially assessed through XRD spectra. The findings (Figure S3) revealed that the crystallization characteristics of MBBs did not exhibit significant changes, and all variants maintained an amorphous structure. Additionally, the Raman spectroscopy was also used for clarifying the carbon structural features of MBBs. As displayed in Figure 1d, the spectra of MBBs revealed two prominent bands at approximately 1350 cm^−1^ (D band) and 1590 cm^−1^ (G band), and the I_D_/I_G_ ratio serves as an indicator to reveal the proportion of defects and disordered structures present in carbon materials. Evidently, the I_D_/I_G_ value increased from 0.83 to 1.08 with the rise in annealing temperature, indicating the formation of more defects with disordered edges on the carbon substrate during the annealing process, the trend of this aligned with the observed changes in BET and V_tot_ [36,37].

The surface chemical compositions of MBBs were assessed through XPS analysis, revealing the presence of C, N, and O elements, as shown in Figure S4a. In contrast to the C 1s spectra of MBB (Figure S4b), which possessed only two peaks at 284.8 and 285.7 eV corresponding to C−C/C=C and C−O/C−N, MBB−x emerged as a new peak at 288.8 eV corresponding to C=O after effective activation [38,39,40]. Significantly, the C=O content (Table S2) exhibited a notable increase from 1.60% to 11.84% as the calcination temperature enhancement, attributed to the facilitated conversion arising from the instability of C−O bonds at elevated temperatures [41]. Moreover, C=O, acting as Lewis basic sites, can also serve as active sites for PMS activation, leading to the generation of ROS [42]. In addition, the contribution of N atoms was weaker than C/O atoms due to the little overall nitrogen content. Additionally, the ratio of pyridine nitrogen to pyridine nitrogen atoms reduced when the calcination temperature increased (Figure S4c) [43]. In the O 1s spectra, a distinctive feature was observed when comparing MBB and MBB-x. Obviously, MBB (Figure S4c) exhibited a single peak at 529.6 eV (O_L_), while MBB-x displayed a novel peak at 531.1 eV, attributed to oxygen adsorbed on the catalyst surface (O_ads_), which was associated with OVs. Crucially, the elevation of calcination temperature resulted in a reduction in total oxygen content (C_total_) and a pronounced increase in the content of O_ads_, indicating the structural defects in BMM-x were formed successfully. These defects were advantageous for the separation and transfer of electrons, thus accelerating the rate of PMS activation to generate ROS [44]. To further affirm the presence of OVs, MBBs were subjected to solid EPR for testifying the number of unpaired electrons in the OVs on the surface of the samples [45,46]. As illustrated in Figure 1e, a distinct harmonic vibrational peak around g = 2.003 was observed for MBB-x, and the intensity of EPR signals significantly escalated from MBB-400 to MBB-800, which suggested a higher content of defective OVs and was consistent with the outcomes obtained from Raman and XPS analyses [47].

### 3.2. Catalytic Performance of MBBs for PMS Activation

**Figure 2:**
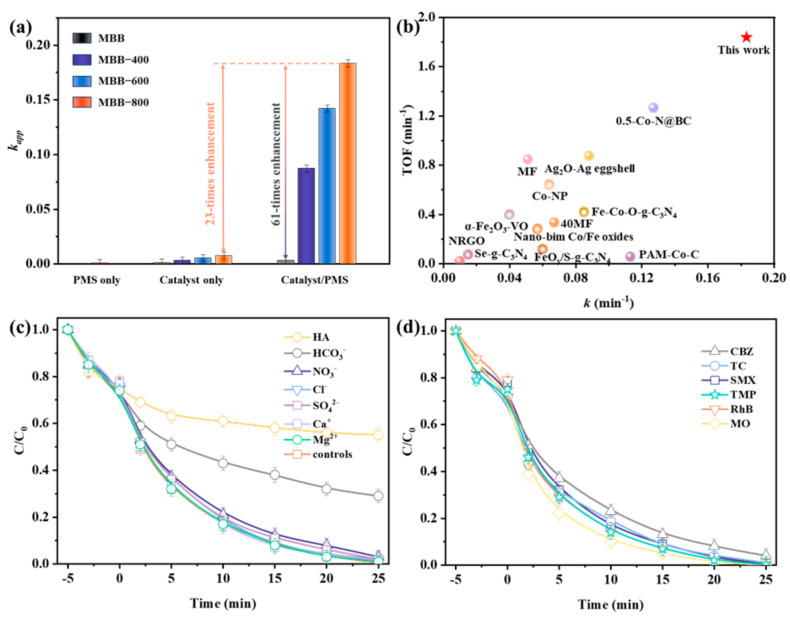
(**a**) Degradation rate of SMX in different systems. (**b**) Relationship of TOF values between different metal-based catalysts and MBB-800 in PMS activation. (**c**) SMX degradation in MBB-800/PMS system under different inorganic anions. (**d**) Degradation of organic pollutants in MBB-800/PMS system. Reaction conditions: [MBB-800] = 0.1 g L^−1^; [oxidants] = 0.33 mM; [organic pollutants] = 1 mg L^−1^; [dyes] = 1 mg L^−1^; pH = 6.8.

The viability of MBBs exhibiting Fenton-like catalytic behavior was further investigated in the context of PMS catalytic decomposition for SMX oxidative degradation. As depicted in Figure S5a, the sharp decrease in SMX concentration within 5 min was mainly attributed to the adsorption of MBB-x, and the adsorption was distributed from 13.58~46.36%. Comparatively, activated MBB-x had a significantly higher adsorption capacity than pristine MBB, and the adsorption efficiency of MBB-x was positively correlated with the calcination temperature, demonstrating that the adsorption capacity depended on the content of OVs in samples [48]. Nonetheless, adsorption alone was not sufficient to entirely eliminate contaminants and may pose the risk of potential secondary contamination. Therefore, it is extremely important to establish a degradation system by introducing oxidizing agents. Obviously, minimal degradation of SMX was observed during sole PMS oxidation, while the simultaneous presence of MBBs and PMS induced a significant enhancement of SMX removal, indicating that PMS alone lacks the capability to generate ROSs for BPA removal (Figure 2a). Similarly, compared with MBB/PMS system, MBB-x/PMS systems exhibited heightened catalytic activity for the removal of SMX, attributed to the abundance of structural defects (OVs) [49]. In particular, the degradation efficiency significantly enhanced with the increase in calcination temperature, and 100% of SMX removal was obtained via the MBB-800/PMS system at 25 min. Meanwhile, the degradation of SMX was well fitted with pseudo-first-order kinetics (ln(C/C_0_) = −*kt*) (Figure S5b), and the rate constant (*k*) of the PMS/MBB-800 system (0.183 min^−1^) was 61 times higher than that of the PMS/MBB system (0.003 min^−1^), visually confirmed the advantage of calcination modification. Moreover, under the PMS/MBB-800 condition, around a 23-fold enhancement of SMX removal rate constant was observed in comparison to MBB-800 alone. Table S3 and Figure 2b included the turnover frequency (TOF), which is derived by dividing the catalyst dosage (mg) by the removal rate for SMX degradation (mg h^−1^) in order to provide a more intuitive assessment of the degradation efficiency [25,50,51,52,53,54,55,56,57,58,59,60]. It was evident that the MBB-800/PMS system demonstrates comparable or even superior performance in degrading SMX compared to the catalysts reported in the literature [61,62]. This implied that the OVs sites and Lewis acid sites in the activated MBB-x acted synergistically to accelerate the electron transfer rate, consequently expediting the conversion rate of PMS to ROSs. Concurrently, the presence of surface OVs served to diminish the reaction barrier for the spontaneous decomposition of PMS, playing a pivotal role in the activation of PMS [44,63], thereby enhancing the degradation performance dramatically. Furthermore, the microporous structure of the defective state and the larger specific surface area contributed to the exposure of an increased number of active sites, providing a partial elucidation for the heightened catalytic activity. Furthermore, achieving a high EPs removal rate with lower catalyst and oxidizer dosages was a significant signal for determining the success of the reaction system. Considering that the PMS/MBB-800 system had relatively significant degradation effect, it is focused on the discussion. The degradation efficiency of SMX increased as expected with increasing PMS or MBB-800 dosage and decreasing initial SMX concentration (Figure S6), and the optimized conditions were found to be [SMX] 0.2 g L^−1^, [MBB-800] 0.1 g L^−1^, and [HSO_5_^−^] 0.033 mM. The PMS/MBB-800 system demonstrated robust pH resilience, substantiated by the negligible alterations in efficiency within the pH range of 2.1–10.2 (Figure S7). Concurrently, the effects of natural organic matter (NOM) on SMX degradation were examined (Figure 2c), elucidating that SO_4_^2−^, Cl^−^, NO_3_^−^, Ca^2+^, and Mg^2+^ exerted negligible inhibition on the degradation of SMX. The introduction of HCO_3_^−^ impeded the degradation efficacy of SMX by obstructing the Lewis acidic site, thereby diminishing active sites and electron transfer between PMS and SMX [46,47]. Moreover, the presence of HA, a commonplace NOM encountered in diverse environmental matrices, curtailed the degradation of SMX, likely attributed to the carboxyl and phenolic hydroxyl groups within HA [64,65]. This characteristic facilitated the adsorption of HA onto the catalyst surface, impeding further interaction between the catalyst’s surface and aqueous pollutants [66,67].

Furthermore, the PMS/MBB-800 system demonstrated outstanding efficacy in tap water (98.0%, Figure S8) and underground water (98.0%), while a slightly reduced removal rate in surface water (75.0%) was influenced by the presence of HCO_3_^−^ and HA, all of these suggesting that it has good application prospects in a practical environment. In order to certify the universal applicability, the degradation of other five diverse medicines and dyes, such as trimethoprim (TMP), tetracycline (TC), carbamazepine (CBZ), rhodamine B (RhB), and methyl orange (MO), were investigated (Figure 2d). All contaminants exhibited remarkable degradation in the MBB-800/PMS system within 25 min, with degradation ratios ranging from 96.1% to 100%. Moreover, the reusability of MBB-800 in consecutive experiments was investigated and depicted in Figure S9, which still showed strong activity for SMX and TMP degradation after five cycles, with 95.3% of SMX and 96.8% of TMP eliminated within 25 min, confirming its good reusability and stability.

### 3.3. Possible Reaction Mechanisms

#### 3.3.1. Identification of Key Reactive Oxygen Species

**Figure 3:**
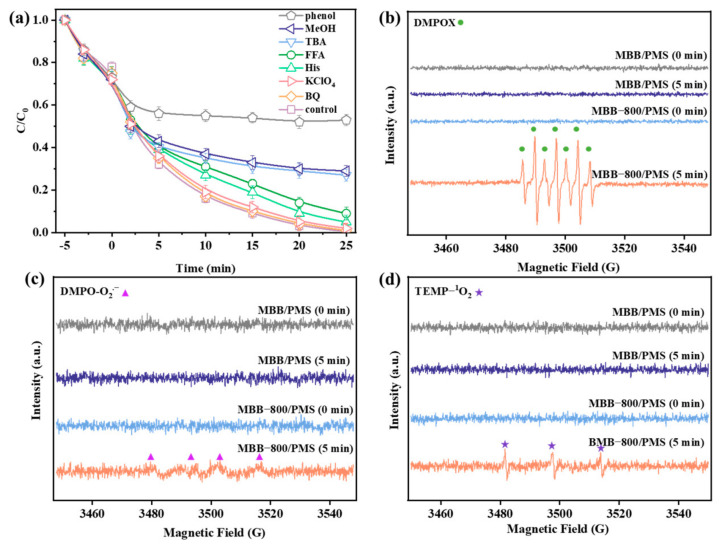
(**a**) The effect of quenching agents of SMX degradation in MBB-800/PMS system. Spin-trapping EPR spectra for (**b**) DMPOX, (**c**) DMPO-O_2_^•−^, and (**d**) TEMP-^1^O_2_ in various systems at different times. Reaction conditions: [MBB-800] = 0.1 g L^−1^; [oxidants] = 0.33 mM; [SMX] = 1 mg L^−1^; pH = 6.8.

To elucidate the degradation mechanism, a free radical quenching experiment was conducted to discern the primary free radical responsible for the degradation of SMX in the PMS/MBB-800 system. MeOH and TBA were employed to differentiate the contributions of SO_4_^•−^/^•^OH and ^•^OH, respectively (K_MeOH+SO4_^•−^ = (1.2–2.8) × 10^7^ M^−1^ s^−1^ [68], K_MeOH+•OH_ = (1.6–7.7) × 10^7^ M^−1^ s^−1^ [68], K_TBA+•OH_ = (3.8–7.6) × 10^8^ M^−1^ s^−1^ [69]). The addition of 1 M MeOH and TBA to the reaction system led to a 39% and 30% decrease (Figure 3a) in the degradation efficiency of SMX, respectively, suggesting that the oxidation process of SMX may be governed by surface-bound SO_4_^•−^/^•^OH species. The introduction of benzoquinone (BQ, BQ, K_BQ+O2_^•−^ = 1.0 × 10^9^ M ^−1^ s ^−1^) [70] had a negligible impact on SMX removal, reaffirming the minimal contribution of O_2_^•−^ in SMX elimination. Furthermore, His (K_His+_^1^_O2_ = 2.3 × 10^7^ M^−1^ s^−1^) [71] and FFA (K_FFA+_^1^_O2_ = 1.2 × 10^8^ M^−1^ s^−1^) [72] were employed to assess the involvement of ^1^O_2_, both of which exhibited less inhibitory effects. These results underscored that the primary radicals involved in the PMS/MBB-800 system were ^•^OH, while ^1^O_2_ and O_2_^•−^ played insignificant roles. In addition, KClO_4_ was utilized to investigate the engagement of the electron transfer pathway mediated by MBB-800-PMS complexes, and the introduction of KClO_4_ marginally hindered the advancement of SMX removal, indicating the dispensability of the facilitated electron transport pathway. Moreover, the surface-bound SO_4_^•−^/^•^OH (SBRs), which was generated and confined on the activator surface, exhibited constrained spatial availability for activity, selectively reacting with SMX adsorbed on the surface or around solution [73,74]. And phenol, known for its inherent hydrophobic properties, was applied as a SBR quencher, selectively contacting the surface of the activator and effectively quenching the SBRs [75,76]. Furthermore, the presence of phenol (150 mM) led to a 46.3% reduction in SMX removal, substantiating that SBRs constituted the predominant reactive oxygen species in the PMS/MBB-800 system.

To substantiate this, an in situ EPR technique was employed to monitor the evolution of involved ROSs generated during PMS activation by MBB and MBB-800, respectively. In the trapping of SO_4_^•−^ and ^•^OH, distinctly from the MBB/PMS system, a characteristic seven-line EPR spectrum (1:2:1:2:1:2:1) was identified in the MBB-800/PMS system (Figure 3b). The synthesis of 5,5-dimethyl-2-oxo-pyrroline-1-oxyl (DMPOX) was attributed to this signal, which was different from the usual signals of DMPO- SO_4_^•−^ and DMPO-^•^OH [77,78]. Prior studies have corroborated the oxidation of DMPO to DMPOX by both free radicals (e.g., SO_4_^•−^ and ^•^OH) and nonradicals (such as high-valent iron-oxo species or ^1^O_2_) at the interface of the catalyst [79,80,81,82]. Considering the results of the aforementioned quenching experiments, there is a reasonable basis to infer that the EPR signal was caused by the further oxidation of DMPO-·OH by ·OH in the bulk solution [83]. Simultaneously, EPR detected only faint signals of O_2_^•−^ (Figure 3c) and ^1^O_2_ (Figure 3d) in the MBB-800/PMS system. The combined results of quenching experiments and EPR suggest that the free radical (^•^OH) and surface-bound SO_4_^•−^/^•^OH significantly contribute to the degradation of SMX. The difference between MBB and MBB-800 can be attributed to the prevalence of OVs, which offer appropriate locations for PMS or O_2_ molecule activation and raise the concentration and transport of surface oxygen species.

#### 3.3.2. Electron Transfer Performance

**Figure 4:**
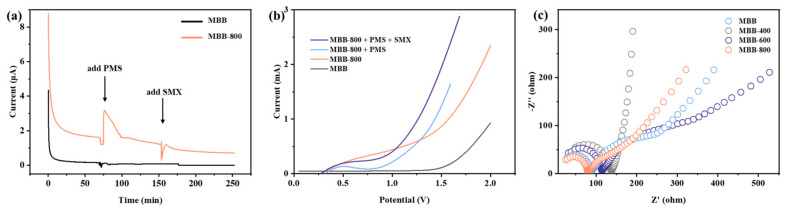
(**a**) Chronoamperometry curve of MBB and MBB-800, (**b**) LSV curves of MBB and MBB-800 in different systems, and (**c**) EIS curves of different samples.

To comprehend the interplay among PMS, SMX, and the catalyst, we employed chronoamperometry, linear sweep voltammetry spectra (LSV), and electrochemical impedance spectroscopy (EIS) to further elucidate the electron transfer pathway. As depicted in Figure 4a, following the sequential introduction of PMS and BMX to the catalyst system, the alteration in the response current of MBB-800 catalyst was notably more pronounced, while there was virtually no change in the response current of MBB. The substantial current surge upon the addition of PMS indicated that MBB-800 acquired electrons from PMS, whereas the introduction of SMX elicited a much weaker current response, signifying a limited current transfer between SMX and MBB-800 [84].

Furthermore, the LSV curves of different catalytic systems were investigated and are presented in Figure 4b. In comparison to pristine MBB, MBB-800 exhibited an enhanced current, indicative of improved electronic transmission performance attributed to the increased OVs. Upon the addition of SMX, the current initially decreased and then increased, as the rapidly adsorbed SMX on the electrode surface concealed active sites such as OVs initially. With the simultaneous addition of PMS and BPA, a noticeable increase in current was observed, which was attributed to the occupation of O atoms in PMS on oxygen vacancies and the formation of activated peroxo species (PMS*), thereby strengthening the electron transfer among MBB-800, PMS, and SMX, consistent with the results in Figure 4a.

Moreover, the EIS arc radius of MBB-800 (Figure 4c) exhibited the smallest semicircle radius in EIS Nyquist plots compared to the other samples under the same external conditions. The decreasing trend correlated with the enhancement in activity, which can be attributed to the OVs enhancing the electron transfer capability of MBB-800 and contributed to the generation of active species, ultimately promoting the activation and effective removal of SMX in the PMS/MBB-800 system [85,86,87].

#### 3.3.3. Possible Degradation Pathways of SMX in PMS/MBB-800 System

**Figure 5:**
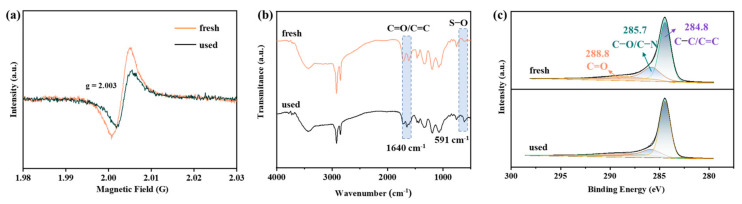
(**a**) EPR spectra, (**b**) FTIR spectra, and (**c**) C 1s XPS spectrum of fresh and used MBB-800.

To gain deeper insights into the enhanced degradation efficiency of SMX by the PMS/MBB-800 system, it was crucial to investigate the content of OVs and key functional groups. In Figure 5a, the decline in OV content after PMS activation suggested that OVs served as active sites in the catalytic activation of PMS to generate ROSs. Additionally, an emergence of a new peak at 1640 cm^−1^ in the FTIR spectra (Figure 5b), attributed to C=O/C=C stretching of aromatic conjugates, was observed in the used MBB-800 [88], potentially originating from the carbonyl groups in rings of intermediates or products, and another new peak emerged around 591 cm^−1^ after the reaction was caused by the S−O stretching of sulfate ions [84]. Moreover, a noticeable decrease in the content of C=O after the SMX degradation reaction, compared to that of fresh MBB-800 (Figure 5c), demonstrated that the C=O group acts as a Lewis base, participating in the cleavage of the O−O bond through electron transfer for PMS activation. Consequently, the C=O groups and oxygen vacancies in MBB-800 can activate the adsorbed HSO_5_^−^ species to produce ^•^OH/SO_4_^•−^ (Equations (1) and (2)), and the ^•^OH can also generate through the reaction of partial SO_4_^•−^ with H_2_O (Equation (3)). In this instance, the PMS compound experienced concurrent oxidation and reduction reactions [89]. Specifically, MBB-800 facilitated the oxidation of PMS at the electron-deficient carbon atom, resulting in the formation of SO_5_^•−^ (Equation (4)), and then engaged in the reaction with H_2_O to the generation of ^1^O_2_ (Equation (5)). Ultimately, SMX can undergo degradation and mineralization by all ROSs, including ^•^OH/SO_4_^•−^/^1^O_2_/SBRs (Equation (6)), originating from the various pathways involved in the process.

OVs + HSO_5_^−^ + e^−^ → OVs^+^ + ^•^OH + SO_4_^2−^ (1)

OVs + HSO_5_^−^ + e^−^ → OVs^+^ + OH^−^ + SO_4_^•−^ (2)

SO_4_^•−^ + H_2_O → ^•^OH + SO_4_^2−^ (3)

HSO_5_^−^ + e^−^ → SO_5_^•−^ + H^+^ (4)

2SO_5_^•−^ + H_2_O → 2HSO_4_^−^ + 1.5^1^O_2_ (5)

^•^OH/SO_4_^•−^/^1^O_2_/SBRs + SMX → intermediate +CO_2_ + H_2_O (6)

**Figure 6:**
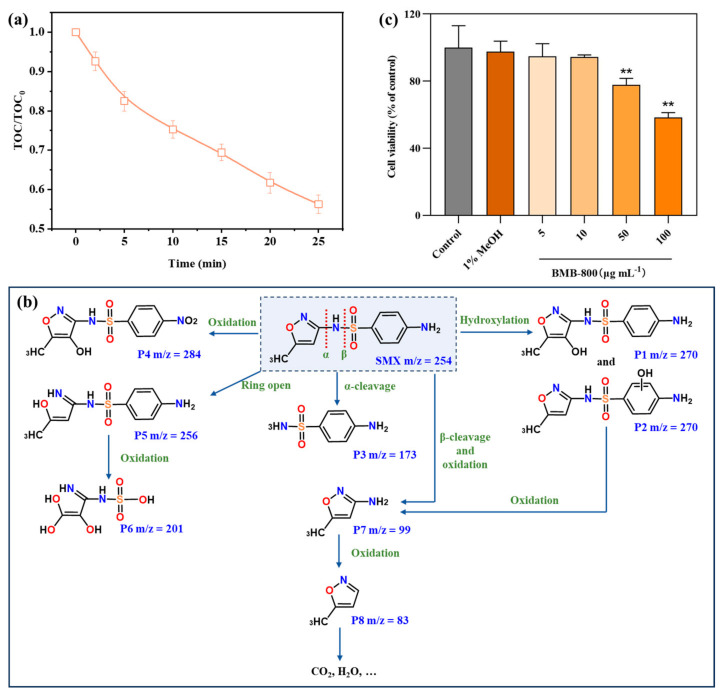
(**a**) TOC removal and (**b**) possible degradation pathways of SMX in MBB-800/PMS system. (**c**) Corresponding cell viability percentage of PC21 cells at the selected concentrations of MBB-800. Data are presented as mean ± SD, ** *p* < 0.01, no significance.

To evaluate the efficacy of the PMS/MBB-800 system in mineralizing SMX, we monitored changes in the concentration of total organic carbon (TOC) in the solution throughout the degradation process, as illustrated in Figure 6a. The results unveiled a significant mineralization, with approximately 43.7% of organic contaminants undergoing transformation into carbon dioxide and water within a 25 min timeframe. This outcome indicated the successful mineralization of a portion of SMX, along with its degraded small molecule organic intermediates.

The intermediate products of SMX were discerned via LC-MS spectra analysis, with the potential degradation pathways illustrated in Figure 6b. The degradation intermediates of SMX in the PMS/MBB-800 system was detailed in Figure S10. Initially, electrophilic replacement occurred on the aromatic ring, resulting in the formation of ortho-hydroxylated SMX (P1). This transformation arises from the electron-withdrawing impact of the sulfonamide group (–SO_2_–NH–) and electron-donating influence of the amine group (–NH_2_). Subsequently, the ortho-derivative concerning the –NH_2_ group, denoted as P2, was obtained. Subsequently, the non-selective hydroxyl radicals targeted the α-bond of sulfamethoxazole, causing the cleavage of the C–N bond, and P3 was produced by the cleaved of nitrogen. Additionally, ^•^OH/SO_4_^•−^ may readily attack the amino group of reactive site N in the benzene ring of SMX, forming the nitro derivative P4 (NO_2_-SMX). Furthermore, the isoxazole ring of SMX was attacked and opened, resulting in P5. The amino group was then oxidized to produce P6. Moreover, sulfamethoxazole’s β-bond was the target of non-selective hydroxyl radicals, leading to the cleavage of the S–N bond. The subsequent removal of a hydrogen by the cleaved nitrogen resulted in the formation of 3-amino-5-methylisoxazole (P7), which was further oxidized to yield P8. Ultimately, these small molecules underwent further mineralization into CO_2_ and H_2_O.

#### 3.3.4. Cytotoxicity of MBB-800

The cell viability of MBB-800 was evaluated using PC12 cells at different MBB-800 concentrations, viz., 5 µg mL^−1^, 10 µg mL^−1^, 50 µg mL^−1^, and 100 µg mL^−1^. As shown in Figure S11, the morphological characteristics of PC12 cells were altered in different concentrations of MBB-800. A microscopic examination revealed consistent cell monolayer architecture and morphological characteristics across all experimental conditions, including controls and treatments with 5 µg mL^−1^ and 10 µg mL^−1^ of MBB-800. However, when the concentration of MBB-800 was further increased by 50 µg mL^−1^ and 100 µg mL^−1^, partial cells twisted into a spherical form. As shown in Figure 6c, the effect of MBB-800 in PC12 cell viability was examined using the CCK8 assay. Cell viability exhibited no significant variance among the control group, as well as the groups treated with 5 µg mL^−1^ and 10 µg mL^−1^ of MBB-800. However, a decrease in cell viability was observed in the groups treated with 50 µg mL^−1^, 100 µg mL^−1^ of MBB-800. These findings suggest that the impact of catalyst dosage on organisms within the MBB-800/PMS system was minimal.

## 4. Conclusions

In synopsis, this investigation successfully synthesized biochar MBB-800 characterized by abundant structural defects (OVs), achieved through a calcination process. The study demonstrated the efficacy of MBB-800 in activating PMS for the degradation of SMX. The PMS/MBB-400 system exhibited efficient SMX degradation over a broad pH range, in the presence of competing ions, and in natural water, demonstrating exceptional recyclability and stability. Notably, MBB-800 displayed versatility in addressing various antibiotics and dyes. Quenching and EPR tests substantiated ^•^OH/SO_4_^•−^ and SBRs as the primary active species responsible for SMX degradation into smaller intermediates. Moreover, through intrinsic characterizations, a substantial distribution of uniform active sites was identified as the pivotal factor for PMS activation, with MBB-800, featuring the highest content of OVs and C=O, exhibiting the swiftest interfacial electron transfer and SMX decomposition efficiency. The investigation also delved into cell viability using the CCK-8 assay, affirming the cytotoxic potential of the prepared materials. These findings not only offer advanced insights into establishing the structure–activity relationships of pristine biochar activators but also present a strategic approach to address practical challenges in wastewater treatment, holding great potential for substantial and sustainable environmental remediation.

## Data Availability

The data presented in this study are available in manuscript.

## Supplementary Materials

The following supporting information can be downloaded at: https://www.mdpi.com/article/10.3390/toxics12040283/s1, Figure S1: TEM images of (a) MBB, (B) MBB-400, and (c) MBB-600; Figure S2: (a) N2 adsorption−desorption isotherms and (b) pore size distribution of different samples; Figure S3: XRD patterns of different samples; Figure S4: (a) Full-range scan, (b) C 1s, (c) N 1s, and (d) O 1s XPS spectrum of different samples; Figure S5: Removal (a) efficiency and (b) kinetics of SMX in different systems; Figure S6: SMX degradation in MBB-800/PMS system under different conditions: (a) catalyst dosage, (b) PMS concentration, (c) SMX concentration. Reaction conditions: [MBB-800] = 0.1 g L^−1^, [PMS] = 0.33 Mm, [SMX] = 1 mg L^−1^, pH = 6.8; Figure S7: SMX degradation in MBB-800/PMS system under different pH value. Reaction conditions: [MBB-800] = 0.1 g L^−1^, [PMS] = 0.33 Mm, [SMX] = 1 mg L^−1^; Figure S8: SMX degradation in MBB-800/PMS system under different water matrices. Reaction conditions: [MBB-800] = 0.1 g L^−1^, [PMS] = 0.33 Mm, [SMX] = 1 mg L^−1^, pH = 6.8; Figure S9: The reusability of degradation SMX and TMP in MBB-800/PMS system. Reaction conditions: [MBB-800] = 0.1 g L^−1^, [PMS] = 0.33 mM, [SMX] = 1 mg L^−1^, [TMP] = 1 mg L^−1^, pH = 6.8; Figure S10: MS spectra of degradation intermediates of SMX in MBB-800/PMS system; Figure S11. Images of PC12 cells under the microscope with different concentration of MBB-800 for 24h. Scale bar = 100 μm; Table S1: Physicochemical properties of different catalysts; Table S2: Peak fitting results of XPS analysis of MBBs; Table S3: The comparison of different catalysts for SMX degradation in PMS activation.
