# Supplementary material for: Effective Activation of Peroxymonosulfate by Oxygen Vacancy Induced Musa Basjoo Biochar to Degrade Sulfamethoxazole: Efficiency and Mechanism
Source: Toxics. 2024 Apr 12;12(4):283. doi: 10.3390/toxics12040283 (PMC11054925; doi:10.3390/toxics12040283)
Supplement: Supplementary file 1 [file toxics-12-00283-s001.zip › toxics-2947990-supplementary.pdf]

Supporting Information

Effective activation of peroxymonosulfate by oxygen vacancy induced Musa basjoo biochar to degradation sulfamethoxazole: efficiency and mechanism

Shuqi Li [1,2], Jian Yang [2], Kaiwen Zheng [2], Shilong He [1], Zhigang Liu [3], Shuang Song [4], and Tao Zeng [4,]*

[1] School of Environment and Spatial Informatics, China University of Mining and Technology, Xuzhou 221116, China

[2] Ecology and Health Institute, Hangzhou Vocational & Technical College, Hangzhou, PR China

[3] Ningbo Water & Environment Group, Ningbo 315100, P.R. China

[4] Key Laboratory of Microbial Technology for Industrial Pollution Control of Zhejiang Province, College of Environment, Zhejiang University of Technology, Hangzhou, Zhejiang, 310032, P.R. China

*** Corresponding Author**

Tao Zeng.

Email: zengtao@zjut.edu.cn; Tel: +86-571-88320726.

Chemicals and reagents

Musa basjoo obtained from Nanning, Guangxi, China. Rhodamine B (RhB), methyl orange (MO), sulfamethoxazole (SMX), Trimethoprim (TMP), carbamazepine (CBZ), tetracycline (TC), tert-butyl alcohol (TBA), 1,4-benzoquinone (BQ), phenol, methanol (MeOH), tert-butyl alcohol (TBA), p-benzoquinone (p-BQ) and furfuryl alcohol (FFA), Potassium perchlorate ($KClO_4$), sodium sulfate ($NaSO_4$), hydrochloride acid (HCl), and sodium hydroxide (NaOH) were purchased from Aladdin Reagent Co. Ltd. (Shanghai, China). peroxymonosulfate (PMS, available as Oxone[@] ($KHSO_5 \cdot 0.5KHSO_4 \cdot 0.5K_2SO_4$)), 5,5-dimethyl-1-pyrroline (DMPO), 2,2,6,6-tetramethyl-4-piperidinol (TEMP, 99%), Foetal Bovine Serum (FBS) and pheochromocytoma (PC12) cells were purchased from Sigma-Aldrich. Differentiated PC12 cells were purchased from Type Culture Collection of Chinese Academy of Sciences. All of the chemicals employed were analytical grade and were used without further purification.

Characterization

The transmission electron microscopy (TEM) images were carried out on an accelerating voltage of 200 kV (Talos-S, FEI, USA). X-ray diffraction (XRD) patterns were performed on a PANalytical X' Pert PRO powder diffractometer using Cu Kα radiation (λ = 0.1541 nm). The fourier transform infrared (FTIR) spectroscopy obtained on a Nexus 670 FTIR spectrometer with KBr as the diluents. X-ray photoelectron spectroscopy (XPS) data were obtained on PerkinElmer PHI 5000 C instrument with a monochromatized Al Kα line source (200 W). Raman spectra were obtained from a LabRAM HR Evolution (HORIBA, Japan) spectrometer. The Brunauer−Emmett−Teller (BET) surface area was obtained from the nitrogen adsorption and desorption isotherms recorded at 77 K using an ASAP 2460 analyzer (Micromeritics, USA). The electron paramagnetic resonance (EPR) measurements were carried out on a Bruker Model A300 spectrometer. The liner sweeps voltammetry (LSV) and electrochemical impedance measurements (EIS) were performed on a CHI 670E electrochemical workstation (CH Instrument, USA) using a three-electrode quartz

cell. The functioning electrode was produced as follows: Photocatalysts (20 mg) were disseminated in isopropanol (300 μL) and 5% Nafion solution (50 μL) for 5 minutes of vigorous oscillations. The suspension (40 μL) was dip-coated on ITO and dried overnight at room temperature. The reference and counter electrodes were Pt flake and Ag/AgCl (saturated KCl), respectively. A 0.1 M aqueous solution of Na_2SO_4 was used as the supporting electrolyte. The cells were cultured in dulbecco's modified eagle medium (DMEM) supplemented with 10% FBS, in a humidified incubator (5% CO_2, 37°C). MBB-800 was dissolved in 1%MeOH. PC12 cells were in 1% MeOH and 5 μg $mL^{-1,}$ 10μg mL^{-1}, 50 μg mL^{-1}, and 100 μg mL^{-1} of MBB-800 for 24 h.

Degradation Experiment

To compare the SMX degradation kinetic rate, the kinetic data were fitted with the pseudo-first-order model as follows:

$$\ln\frac{C_0}{C_t}=Kt \tag{S1}$$

where C_0 is the initial solution concentration (mg L^{-1}), C_t is the concentration at time t (mg L^{-1}), and K (min^{-1}) is the rate constant for the pseudo-first-order kinetic models. Further, the effect of the initial pH was investigated by adjusting the pH to 2.1–10.2 using 0.01 M NaOH or HCl.

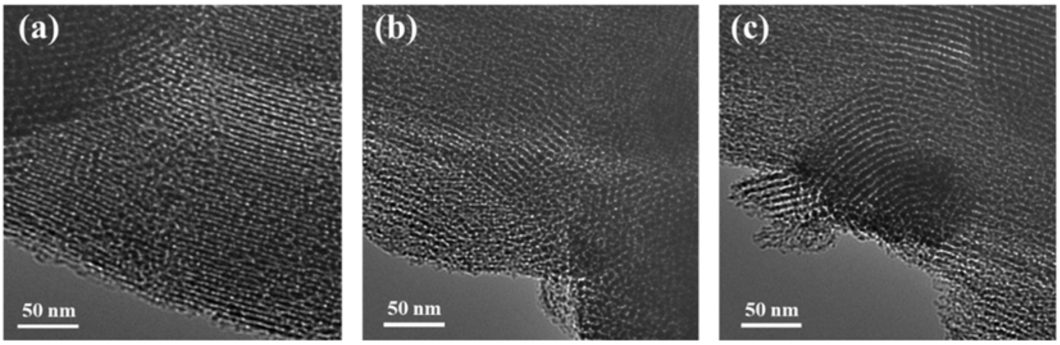

Figure S1. TEM images of (a) MBB, (B) MBB-400, and (c) MBB-600.

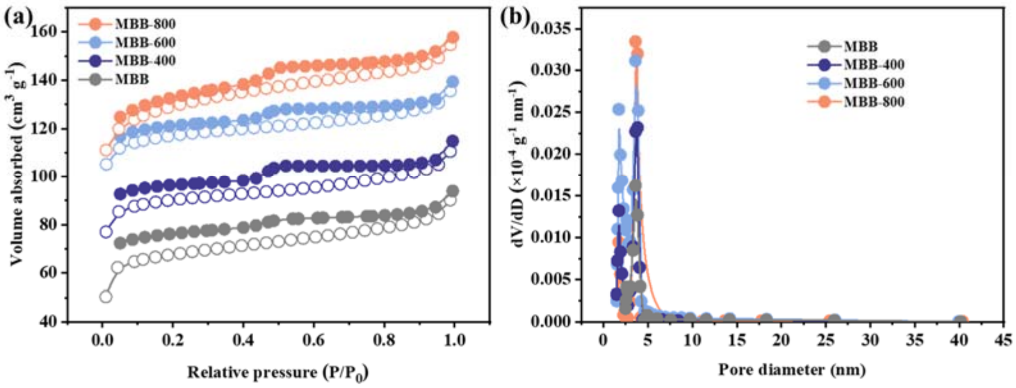

Figure S2. (a) N_2 adsorption−desorption isotherms and (b) pore size distribution of different samples.

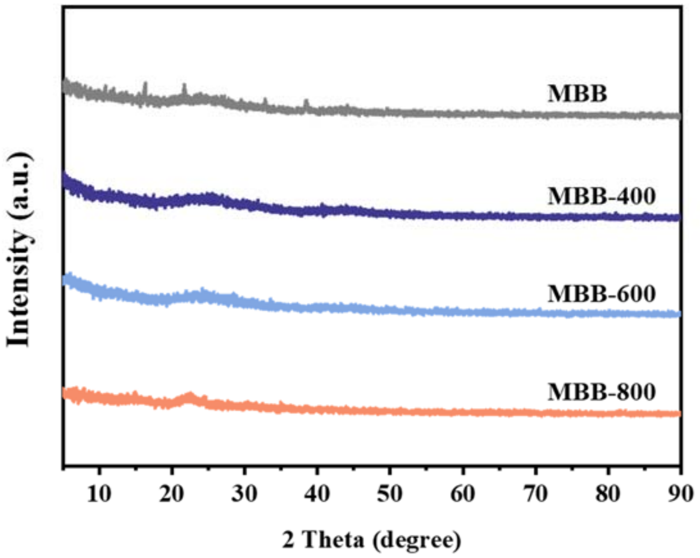

Figure S3. XRD patterns of different samples.

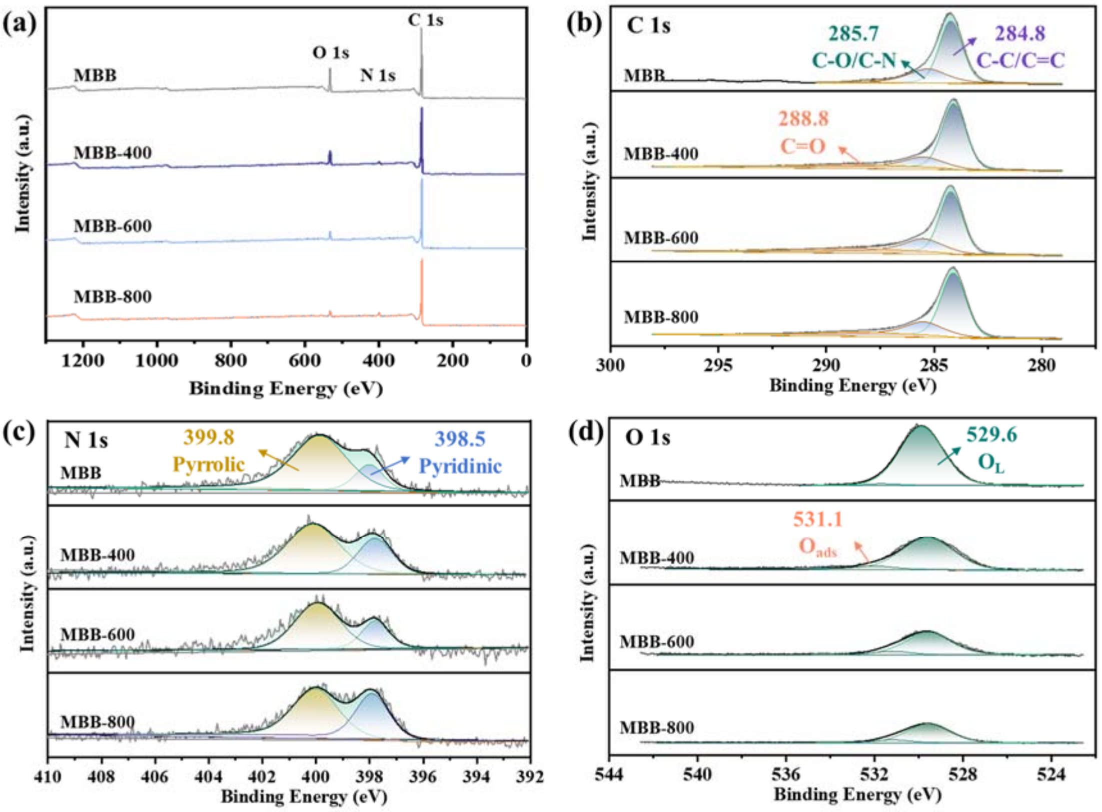

Figure S4. (a) Full-range scan, (b) C 1s, (c) N 1s, and (d) O 1s XPS spectrum of different samples.

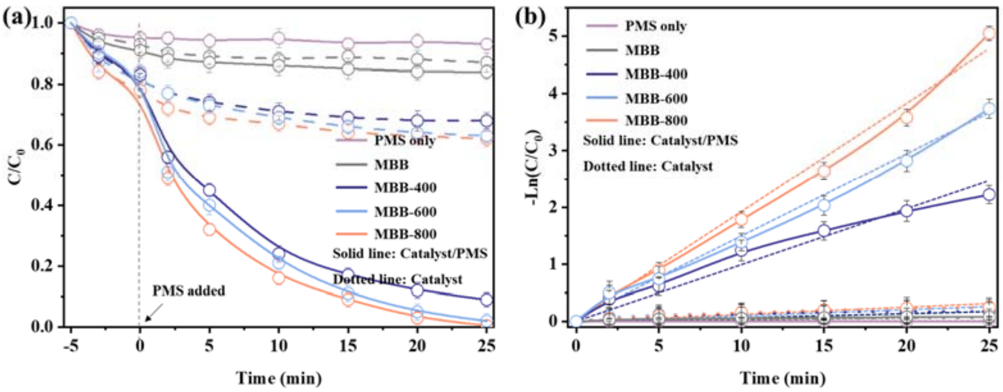

Figure S5. Removal (a) efficiency and (b) kinetics of SMX in different systems.

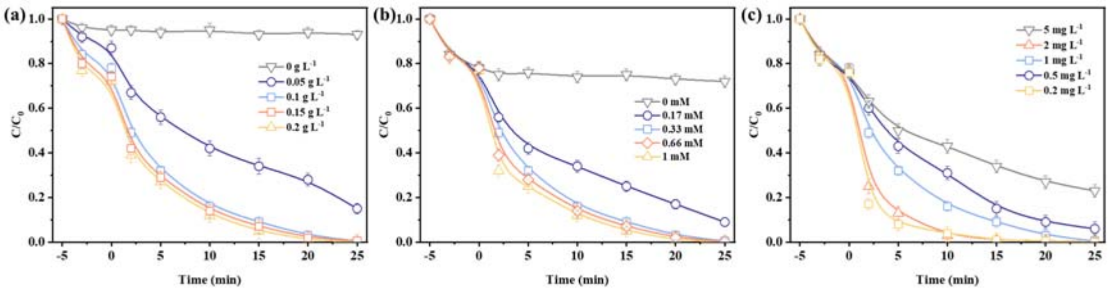

Figure S6. SMX degradation in MBB-800/PMS system under different conditions: (a) catalyst dosage, (b) PMS concentration, (c) SMX concentration. Reaction conditions: [MBB-800] = 0.1 g L^{-1}, [PMS] = 0.33 Mm, [SMX] = 1 mg L^{-1}, pH = 6.8.

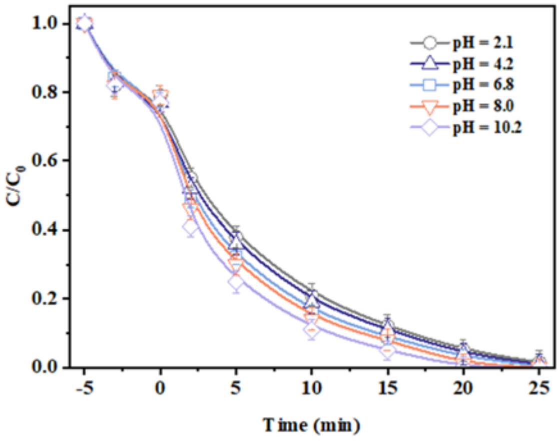

Figure S7. SMX degradation in MBB-800/PMS system under different pH value. Reaction conditions: [MBB-800] = 0.1 g L^{-1}, [PMS] = 0.33 Mm, [SMX] = 1 mg L^{-1}.

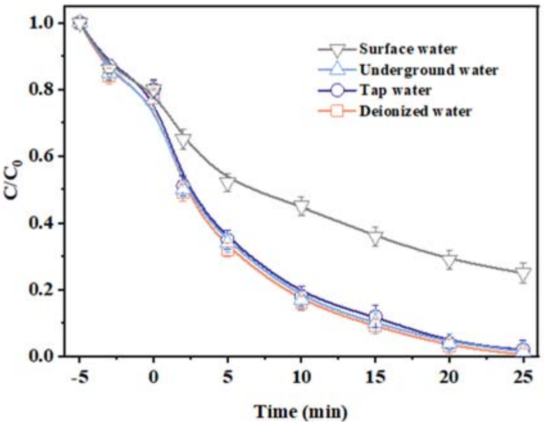

Figure S8. SMX degradation in MBB-800/PMS system under different water matrices. Reaction conditions: [MBB-800] = 0.1 g L^{-1}, [PMS] = 0.33 Mm, [SMX] = 1 mg L^{-1}, pH = 6.8.

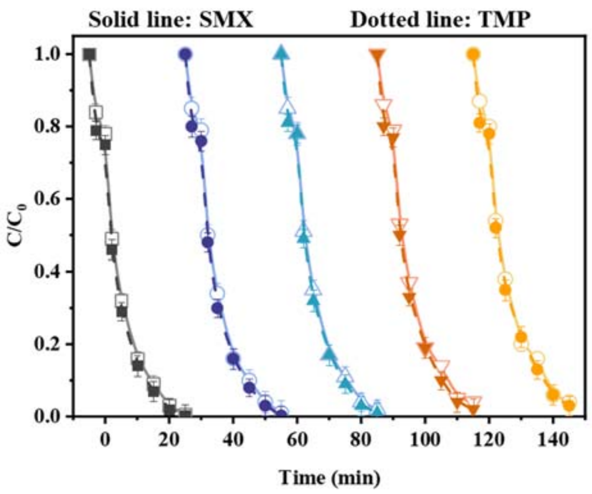

Figure S9. The reusability of degradation SMX and TMP in MBB-800/PMS system. Reaction conditions: [MBB-800] = 0.1 g L^{-1}, [PMS] = 0.33 mM, [SMX] = 1 mg L^{-1}, [TMP] = 1 mg L^{-1}, pH = 6.8.

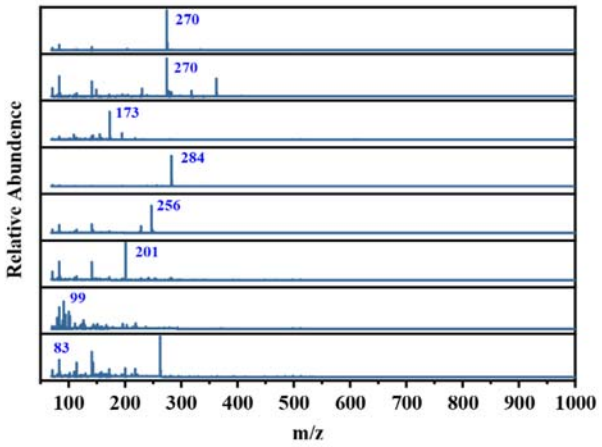

Figure S10. MS spectra of degradation intermediates of SMX in MBB-800/PMS system.

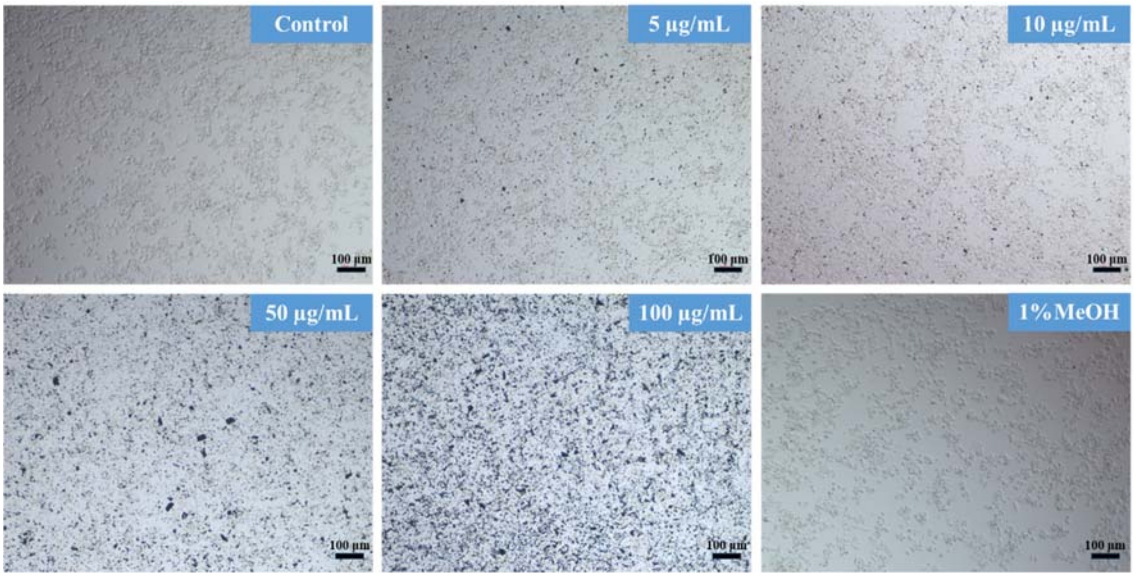

Figure S11. Images of PC12 cells under the microscope with different concentration of MBB-800 for 24h. Scale bar =100 μm.

Table S1 Physicochemical properties of different catalysts.

Catalyst	Total pore volume (V_{tot}) $cm^3\ g^{-1}$	Micropore volume (V_{mic}) $cm^3\ g^{-1}$	BET **surface area** (S_{BET}) $m^2\ g^{-1}$
MBB	0.1453	0.0848	260.7
MBB-400	0.1776	0.1250	348.5
MBB-600	0.2156	0.1651	451.4
MBB-800	0.2441	0.1666	491.4

Table S2 Peak fitting results of XPS analysis of MBBs.

Catalyst	Atomic Concentrationa (%)						
	C 1s				O 1s		
	C_{total}	C−C/C=C	C−O/C−N	C=O	O_{total}	O_L	O_{ads}
MBB	83.70	58.64	23.47	1.60	15.23	15.23	0
MBB-400	90.08	61.13	20.57	8.37	6.87	6.46	0.41
MBB-600	92.61	57.92	25.59	9.10	4.75	4.17	0.58
MBB-800	94.25	55.91	26.50	11.84	3.89	3.30	0.59

Table S3 The comparison of different catalysts for SMX degradation in PMS activation.

Catalysts	SMX (μm)	Catalyst dosage (g L^{-1})	PMS (mM)	Degradation rate (%)	*k* (min^{-1})	TOF (min^{-1})	Ref.
NRGO	40.0	0.5	0.80	91.7	0.010	0.020	[1]
PAM-Co-C	39.5	2.0	0.33	99.3	0.113	0.057	[2]
Se-g-C_3N_4	39.5	0.2	1.30	93.0	0.015	0.075	[3]
FeO_y/S-g-C_3N_4	40.0	0.5	0.80	100	0.060	0.120	[4]
Nano-bim Co/Fe oxides	40.0	0.2	0.40	100	0.057	0.285	[5]
40MF	40.0	0.1	0.16	92.9	0.067	0.335	[6]
α-Fe_2O_3-VO	10	0.1	0.1	91.5	0.040	0.403	[7]
Fe-Co-O-g-C_3N_4	40.0	0.2	0.80	100	0.085	0.425	[8]
Co-NP	9.9	0.1	0.15	100	0.064	0.644	[9]
MF	40.0	0.06	0.81	90.0	0.051	0.848	[10]
Ag_2O-Ag eggshell	40.0	0.1	0.16	94.7	0.088	0.877	[11]
0.5-Co-N@BC	40.0	0.1	0.40	99.6	0.127	1.267	[12]
**SA Co(24)-N/C catalyst**	**4.0**	**0.1**	**0.33**	**100**	**0.183**	**1.830**	**This work**

Reference

1. Wang, S.; Xu, L.; Wang, J. Nitrogen-Doped Graphene as Peroxymonosulfate Activator and Electron Transfer Mediator for the Enhanced Degradation of Sulfamethoxazole. *Chem. Eng. J.* **2019**, *375*, 122041, doi:10.1016/j.cej.2019.122041.
2. Bao, Y.; Shan, Y.; Lim, T.; Wang, R.; David, R. Polyacrylonitrile (PAN) - Induced Carbon Membrane with in-Situ Encapsulated Cobalt Crystal for Hybrid Peroxymonosulfate Oxidation- Fi Ltration Process : Preparation , Characterization and Performance Evaluation. *Chem. Eng. J.* **2019**, *373*, 425–436, doi:10.1016/j.cej.2019.05.058.
3. Tian, Y.; Tian, X.; Zeng, W.; Nie, Y.; Yang, C.; Dai, C.; Li, Y.; Lu, L. Enhanced Peroxymonosulfate Decomposition into ·OH and $^1O_2$ for Sulfamethoxazole Degradation over Se Doped g-C_3N_4 Due to Induced Exfoliation and N Vacancies

Formation. *Sep. Purif. Technol.* **2021**, *267*, 118664, doi:10.1016/j.seppur.2021.118664.

4. Wang, S.; Liu, Y.; Wang, J. Iron and Sulfur Co-Doped Graphite Carbon Nitride ($FeOy/S-g-C_3N_4$) for Activating Peroxymonosulfate to Enhance Sulfamethoxazole Degradation. *Chem. Eng. J.* **2020**, *382*, 122836, doi:10.1016/j.cej.2019.122836.

5. Bao, Y.; Oh, W.; Lim, T.; Wang, R.; David, R.; Hu, X. Elucidation of Stoichiometric Efficiency, Radical Generation and Transformation Pathway during Catalytic Oxidation of Sulfamethoxazole via Peroxymonosulfate Activation. *Water Res.* **2019**, *151*, 64–74, doi:10.1016/j.watres.2018.12.007.

6. Xu, X.; Lin, R.; Deng, X.; Liu, J. In Situ Synthesis of FeOOH-Coated Trimanganese Tetroxide Composites Catalyst for Enhanced Degradation of Sulfamethoxazole by Peroxymonosulfate Activation. *Sep. Purif. Technol.* **2021**, *275*, 119184, doi:10.1016/j.seppur.2021.119184.

7. Qin, Q.; Liu, T.; Zhang, J.; Wei, R.; You, S.; Xu, Y. Facile Synthesis of Oxygen Vacancies Enriched α-Fe_2O_3 for Peroxymonosulfate Activation: A Non-Radical Process for Sulfamethoxazole Degradation. *J. Hazard. Mater.* **2021**, *419*, 126447, doi:10.1016/j.jhazmat.2021.126447.

8. Wang, S.; Liu, Y.; Wang, J. Peroxymonosulfate Activation by Fe-Co-O-Codoped Graphite Carbon Nitride for Degradation of Sulfamethoxazole. *Environ. Sci. Technol.* **2020**, *54*, 10361–10369, doi:10.1021/acs.est.0c03256.

9. Liu, F.; Zhou, H.; Pan, Z.; Liu, Y.; Yao, G.; Guo, Y.; Lai, B. Degradation of Sulfamethoxazole by Cobalt-Nickel Powder Composite Catalyst Coupled with Peroxymonosulfate : Performance , Degradation Pathways and Mechanistic Consideration. *J. Hazard. Mater.* **2020**, *400*, 123322, doi:10.1016/j.jhazmat.2020.123322.

10. Guo, R.; Wang, Y.; Li, J.; Cheng, X.; Dionysiou, D.D. Applied Catalysis B : Environmental Sulfamethoxazole Degradation by Visible Light Assisted Peroxymonosulfate Process Based on Nanohybrid Manganese Dioxide Incorporating Ferric Oxide. *Appl. Catal. B Environ.* **2020**, *278*, 119297, doi:10.1016/j.apcatb.2020.119297.

11. Gao, Y.; Zhao, Q.; Li, Y.; Li, Y.; Gou, J.; Cheng, X. Degradation of Sulfamethoxazole by Peroxymonosulfate Activated by Waste Eggshell Supported Ag $_2$O-Ag Nano–Particles. *Chem. Eng. J.* **2021**, *405*, 126719, doi:10.1016/j.cej.2020.126719.
12. Gu, C.; Zhang, Y.; He, P.; Zhu, J.; Gan, M. Insights into Biochar Supported Atomically Dispersed Cobalt as an Efficient Peroxymonosulfate Activator for Sulfamethoxazole Degradation: Robust Performance, ROS and Surface Electron-Transfer Pathways. *Environ. Sci. Nano* **2022**, *9*, 3551–3561, doi:10.1039/d2en00490a.
